# Prognostic significance of CD44s expression in oral squamous cell carcinoma: An immuno-histo-chemical analysis

**DOI:** 10.6026/973206300214060

**Published:** 2025-11-15

**Authors:** Hershita Singh, Sandeep Sidhu, Arun Bhardwaj, Arpit Sharma, Mamta Sharma, Shivendra Rana

**Affiliations:** 1 Department of Oral Pathology and Microbiology, National Dental College, DeraBassi, Mohali, Punjab, India; 2 Department of Oral Pathology and Microbiology, Maharaja Ganga Singh Dental College and Research Centre, Sriganganagar, Rajasthan, India; 3 Department of Oral Pathology and Microbiology, Swami Devi Dyal Hospital & Dental College, Golpura, Barwala, Panchkula, India; 4 Department of Medical Administration, Homi Bhabha Cancer Hospital, Sangrur, Punjab, India; 5 Department of Oral Pathology and Microbiology, Rajasthan Dental College and Hospital, Jaipur, Rajasthan, India; 6 Department of Oral Pathology and Microbiology, Rajasthan Dental College and Hospital, Jaipur, Rajasthan, India

**Keywords:** Oral squamous cell carcinoma, CD44s, immunohistochemistry, prognostic marker, lymph node metastasis, tumor differentiation, clinical stage

## Abstract

Oral squamous cell carcinoma (OSCC) is a common malignancy with a poor prognosis, highlighting the need for reliable molecular biomarkers. CD44s, a cell adhesion molecule, has been implicated in tumor progression, but its prognostic role in OSCC is unclear. This retrospective study evaluated CD44s expression in 50 OSCC cases using immunohistochemistry and an immunoreactive scoring system. High CD44s expression significantly correlated with advanced stage, lymph node metastasis and poor histological grade. CD44s overexpression may serve as a prognostic biomarker to identify high-risk OSCC patients requiring aggressive therapy.

## Background:

The oral squamous cell carcinoma (OSCC) constitutes more than 90 percent of all oral malignancies and is a significant health issue in the world, with 377,000 new cases and 177,000 deaths each year [1]. Nevertheless, the overall survival rate of patients diagnosed with OSCC has been dismal, with only a range of 50-60 years despite the improvement in treatment modalities, such as surgery, radiation and chemotherapy [2]. These risk factors are all established and they include tobacco use, alcohol intake and high-risk human papillomavirus (HPV) infection [1]. The present gold standard system in the prognostication of OSCC is the American Joint Committee on Cancer (AJCC) TNM staging system, which relies on the size of the tumor (T), involvement of lymph nodes in the area (N) and distant metastasis (M) [3]. Although essential, the TNM has weaknesses because it fails to consider the inherent biological differences of tumors. A single patient may vary in clinical outcome significantly with individuals with similar clinical stage and hence, there is a dire need to identify molecular biomarkers that may be a better predictor of tumor behavior, responsiveness to treatment and patient survival [4]. As part of the hunt to identify such biomarkers, efforts have shifted to those molecules that are associated with important cancer-related events such as cell adhesion, migration and invasion. Such a candidate is CD44, which is a multifunctional, single-pass transmembrane glycoprotein. CD44 is a complex gene and it can form as many as many isoforms by alternative splicing [5]. The standard form, CD44s, is the most widespread and most frequently expressed form of the isoform, which does not contain any variant exons. CD44s is the main cell surface receptor of hyaluronan (HA), which is one of the major components of the extracellular matrix. CD44 and HA interaction plays an important role in the regulation of cell-cell and cell-matrix interactions, cell proliferation and cell motility [6]. New evidence has linked CD44 to the pathogenesis and progression of different malignancies. It is generally considered to be a cancer stem cell (CSCs), a specific group of tumor cells that tumors grow, sustain, metastasize and become resistant to therapy [7].

HA binding to CD44 may initiate intracellular signaling cascades, such as the RhoGTPase pathway, the PI3K/Akt pathway, in favor of mechanisms that promote tumor progression, such as epithelial-mesenchymal transition (EMT) [8]. Nonetheless, the exact role and prognostic importance of the CD44s isoform may also dramatically differ among the types of cancers. Although recent discoveries in breast and gastric cancer indicated that high expression of CD44s is associated with low prognosis [9], contradicting studies have been carried out in colorectal cancer. The importance of CD44 expression is yet to be understood in the context of OSCC. A few studies have examined the importance of total CD44 or its isoforms (e.g., CD44v6) with inconclusive results in most cases [10]. Individual contribution of the standard isoform, CD44s, that forms the backbone of all isoforms, has not been studied in isolation to such a degree. To prove its potential as a good prognostic parameter, a clear picture of its correlation with clinicopathological parameters in OSCC is needed. Consequently, the current research aimed to examine the immunohistochemical pattern in the expression of CD44s in a well-characterized cohort of cases of OSCC and to define its relationship with the prognostic indicators such as clinical stage, lymph node status and histopathological grade, which are already known to predict cancer outcome.

## Materials and Methods:

## Study design and sample collection:

This retrospective, cross-sectional study was conducted on 50 archival formalin-fixed, paraffin-embedded (FFPE) tissue blocks of primary OSCC diagnosed between January 2018 and December 2021.

## Inclusion and exclusion criteria:

Inclusion criteria were: (1) histopathologically confirmed cases of primary OSCC, (2) availability of complete clinicopathological data (age, gender, tumor site, TNM stage and histopathological grade), (3) sufficient tissue in the FFPE block for immunohistochemical analysis and (4) patients who underwent primary surgical resection as the initial treatment. Exclusion criteria were: (1) recurrent or metastatic tumors, (2) patients who had received neoadjuvant chemotherapy or radiotherapy, (3) cases with incomplete clinical records and (4) FFPE blocks with poor tissue preservation or processing artifacts.

## Immunohistochemistry (IHC):

Four-micrometer-thick sections were cut from each FFPE block and mounted on poly-L-lysine-coated slides. The sections were deparaffinized in xylene and rehydrated through a graded series of ethanol. Heat-induced antigen retrieval was performed by immersing the slides in a 10 mM citrate buffer (pH 6.0) in a pressure cooker for 15 minutes. Endogenous peroxidase activity was blocked by incubating the sections with 3% hydrogen peroxide in methanol for 20 minutes. After washing with phosphate-buffered saline (PBS), non-specific binding was blocked using 5% bovine serum albumin (BSA) for 30 minutes. The sections were then incubated overnight at 4°C with a mouse monoclonal primary antibody against CD44s (Clone: DF1485, Dako, Glostrup, Denmark) at a dilution of 1:100. Following washes in PBS, the slides were incubated with a horseradish peroxidase (HRP)-conjugated polymer-based secondary antibody (EnVision™ Detection System, Dako) for 30 minutes at room temperature. The immunoreaction was visualized using 3,3'-diaminobenzidine (DAB) as the chromogen. Finally, the sections were counterstained with Harris's hematoxylin, dehydrated, cleared and mounted. Normal human tonsil tissue, known to express CD44, served as the positive control. The negative control was obtained by omitting the primary antibody during the incubation step.

## Evaluation of immunohistochemical staining:

All stained slides were independently evaluated by two experienced pathologists who were blinded to the clinical data. Any discrepancies were resolved by consensus discussion. CD44s expression was assessed based on a semi-quantitative Immunoreactive Score (IRS).

The IRS was calculated by multiplying the score for staining intensity by the score for the percentage of positive tumor cells.

[1] Staining Intensity Score: 0 (no staining), 1 (weak), 2 (moderate) and 3 (strong).

[2] Percentage of Positive Cells Score: 0 (0% positive cells), 1 (<10%), 2 (10-50%) and 3 (>50%).

The final IRS for each case ranged from 0 to 9 (Intensity Score x Percentage Score). Based on the median IRS value of the cohort (Median IRS = 6), cases were dichotomized into two groups: 'Low CD44s expression' (IRS ≤ 6) and 'High CD44s expression' (IRS > 6).

## Statistical analysis:

All data were compiled and analyzed using SPSS Statistics for Windows, Version 25.0 (IBM Corp., Armonk, NY). Descriptive statistics (mean, standard deviation, frequency and percentage) were used to summarize the data. The Chi-square test or Fisher's exact test was used to assess the correlation between categorical variables (CD44s expression groups vs. clinicopathological parameters). Student's t-test was used to compare the mean IRS between two groups. A p-value of less than 0.05 was considered statistically significant.

## Results:

The study included 50 patients with OSCC. The mean age of the patients was 58.6 ± 11.2 years (range: 35-79 years). The cohort comprised 34 males (68.0%) and 16 females (32.0%). The most common tumor site was the tongue (44.0%), followed by the buccal mucosa (30.0%). According to the AJCC 8th edition staging, 23 cases (46.0%) were in early clinical stages (I/II) and 27 cases (54.0%) were in advanced stages (III/IV). Histopathologically, 15 cases (30.0%) were well-differentiated, 22 (44.0%) were moderately differentiated and 13 (26.0%) were poorly differentiated. Lymph node metastasis was present in 26 cases (52.0%). The detailed clinicopathological characteristics are presented in Table 1 (see PDF). Immuno-reactivity for CD44s was predominantly observed as a brown precipitate on the cell membrane, with some cases also showing cytoplasmic staining. The expression was heterogeneous, both between and within tumors. In many cases, staining was most intense in the tumor cells at the invasive front. Of the 50 cases, 22 (44.0%) were classified as having Low CD44s expression (IRS ≤ 6) and 28 (56.0%) had High CD44s expression (IRS > 6). The correlation between CD44s expression status and various clinicopathological parameters is summarized in Table 2 (see PDF). High CD44s expression was significantly associated with advanced clinical stage. Among the 28 cases with high CD44s, 20 (71.4%) were stage III/IV, whereas only 7 (31.8%) of the 22 cases with low CD44s were in advanced stages (p=0.008). A highly significant correlation was observed with lymph node status. Positive lymph node metastasis was found in 21 of 28 cases (75.0%) with high CD44s expression, compared to only 5 of 22 cases (22.7%) with low CD44s expression (p=0.003). Furthermore, CD44s expression correlated significantly with the histopathological grade of the tumor. High CD44s expression was observed in 11 of 13 poorly differentiated tumors (84.6%), while it was present in only 17 of 37 well/moderately differentiated tumors (45.9%) (p=0.012). No significant correlation was found between CD44s expression and patient age, gender, or tumor site (p>0.05). To further quantify these associations, the mean IRS was compared across different clinicopathological groups (Table 3 - see PDF). The mean IRS in tumors with positive lymph node metastasis (7.9 ± 2.1) was significantly higher than in tumors without metastasis (4.3 ± 1.8; p<0.001). Similarly, the mean IRS for advanced-stage (III/IV) tumors (7.5 ± 2.3) was significantly higher than for early-stage (I/II) tumors (4.8 ± 2.0; p<0.001). When stratified by grade, poorly differentiated tumors exhibited a significantly higher mean IRS (8.2 ± 1.9) compared too well and moderately differentiated tumors combined (5.1 ± 2.4; p<0.001).

## Discussion:

The management of OSCC is hampered by the unpredictable clinical behavior of tumors, underscoring the need for reliable prognostic biomarkers. This study investigated the expression of the standard CD44 isoform, CD44s, in OSCC and its association with established clinicopathological parameters. Our principal finding is that high expression of CD44s is significantly correlated with indicators of poor prognosis, including advanced clinical stage, positive lymph node metastasis and poor histopathological differentiation. The biological functions of CD44 provide a strong rationale for these findings. As the primary receptor for hyaluronan, CD44s plays a pivotal role in mediating cell-matrix adhesion and signaling. Overexpression of CD44 can enhance tumor cell motility and invasion by facilitating cellular attachment to HA-rich extracellular matrices and by triggering downstream signaling that promotes cytoskeletal rearrangements [8]. The observation of strong staining of CD44s on the invasive front of tumors agrees with this process and it is supposed that the cells carrying CD44s are actively involved in the invasion of tissues. This is a precondition to the expansion of the tumor locally as well as in distant metastases [11]. A notably significant finding of our study is the high level of association between high expression of CD44s and metastasis in lymph nodes (p=0.003). The involvement of lymph nodes is regarded as one of the strongest predictors of low survival rates among OSCC patients [3]. We can propose that in the metastatic cascade, CD44s can be a key molecule. This is in line with other past studies in other cancers that have demonstrated that the CD44-expressing cells have increased migratory and invasive capacity [12]. Interaction of CD44 with other molecules, e.g., matrix metalloproteinases (MMPs), can also enhance degradation of the basement membrane, thereby helping tumor cells to enter lymphatic or blood vessels [13, 14-15]. Furthermore, we found a significant link between high CD44s expression and poor histopathological grade (p=0.012). Poorly differentiated tumors, characterized by a loss of normal cellular architecture and high mitotic activity, are inherently more aggressive. This correlation may be explained by the role of CD44 in the EMT, a process where epithelial cells lose their polarity and acquire a mesenchymal phenotype, which is associated with dedifferentiation, invasion and stemness [8, 14]. The CD44-positive cell population may represent a more undifferentiated, stem-like subpopulation within the tumor, which is known to be more aggressive and resistant to therapy [7, 16 and 17]. These discrepancies could be attributed to differences in study methodologies, including the specific antibodies used (which may recognize different epitopes or isoforms), variations in scoring systems, different patient cohorts and the inherent complexity of CD44 biology. Our study contributes to this field by specifically focusing on the standard CD44s isoform, providing clearer evidence for its role in OSCC progression. This study has several limitations. First, its retrospective design and relatively small sample size from a single institution may limit the generalizability of our findings. Second, we did not perform survival analysis, which is crucial for definitively establishing a molecule's prognostic value. Third, immunohistochemistry is a semi-quantitative method and while we used a standardized scoring system, it is subject to inter-observer variability. Future research should involve larger, multi-center prospective studies with long-term follow-up to validate our findings and correlate CD44s expression directly with patient survival outcomes. Additionally, employing quantitative digital pathology and molecular techniques like qPCR or RNA-seq could provide more objective measurements of CD44s expression.

## Conclusion:

High expression of the standard CD44 isoform (CD44s) is a frequent event in oral squamous cell carcinoma and is significantly associated with aggressive clinicopathological features. These findings strongly suggest that CD44s plays a critical role in the progression and metastatic spread of OSCC. Although further validation in larger prospective cohorts is required, CD44s emerges as a promising prognostic biomarker.
